# Metacognitive regulation in physical therapists’ clinical reasoning: a scoping review

**DOI:** 10.5116/ijme.6a0c.4c0b

**Published:** 2026-06-03

**Authors:** Yannick Perdrix, Jérémy Nael, Eric Dionne, Nicolas Pinsault

**Affiliations:** 1 Laboratory TIMC (UMR CNRS 5525), Faculty of Medecine, University of Grenoble Alpes, Grenoble, France; 2 Faculty of Education, University of Ottawa, Ontario, Canada

**Keywords:** Metacognitive regulation, reflexivity, clinical reasoning, physical therapy, uncertainty

## Abstract

**Objectives:**

The objective of this scoping review was to explore: (1) the nature and purposes of metacognitive regulation in clinical reasoning activities in Physical Therapy, and (2) the breadth and diversity of methods and tools used to assess metacognitive regulation in clinical reasoning activities in Physical Therapy.

**Methods:**

The nine steps of the JBI guideline and the Prisma-Scr checklist were used. The Pubmed, Google Scholar, PsycINFO, EBSCO, and ERIC databases were queried. Resources published after 2000, in English and French, were searched using a broad range of terms defining metacognitive regulation. Two researchers carried out the parallel and blinded selection and extraction of data concerning the nature of metacognitive regulation (timing, type, task characteristics) and its purposes, as well as assessment methods and tools.

**Results:**

Of the 1,025 identified articles, 36 were analyzed. An integrative model was created, synthesizing the relationships between five key characteristics: the type, timing, task, focal, interaction, and purposes of metacognitive regulation. Assessment methods showed diversity, while remaining underdeveloped overall.

**Conclusions:**

This research formalizes a conceptual framework specific to Physical Therapists metacognitive regulation during clinical reasoning. It establishes a framework and synthesizes existing tools, opening up possible avenues to clarify and develop metacognitive regulation, or engage in research in the field of education.

## Introduction

Clinical reasoning (CR), the cornerstone of patient care, has been the subject of a vast field of research. It has been considered a cognitive, situated and socially mediated activity.^1^ It has evolved over time, with different models,^2^^–^^4^ highlighting different types of reasoning strategies,^5^ reasoning approaches,^6^^,^^7^ mindfulness, communication, values and metacognition. ^6^^–^^8^ More specifically for physical therapists (PT), Huhn and colleagues^9^ define clinical reasoning as a contextual, adaptive, iterative and collaborative process, integrating cognitive, psychomotor and affective skills and involving both the therapist’s and the patient's perspectives in a biopsychosocial approach. They also highlight the specific use of PTs’ and patients’ bodies as key information gathering and transmission components of CR in PTs’ practice, and the role of movement scripts as a specialized form of practice-derived knowledge used in clinical reasoning, as well as metacognition by the clinician.

Metacognition refers to the awareness of one’s own thinking, the content of one’s conceptions,^10^ and the ability to control and regulate cognitive processes.^11^

It consists of^10^^,^^12^^,^^13^ i) knowledge about one's own cognitive abilities, about tasks (declarative, procedural, and conditional knowledge), and about goals; ii) metacognitive experiences including metacognitive sensations and judgments; iii) metacognitive regulation of processes. This regulation is the operational process of metacognition, which comprises two major and complementary processes: monitoring, which corresponds to a self-assessment of one's processes, and control, which relies on monitoring to enable the adjustment of processes (giving up the activity, maintaining or changing strategy, allocating new resources, etc.). These processes are sometimes subdivided into three processes,^14^ i.e., planning, monitoring, and control, or five sub-processes,^15^ adding information management and debugging.

Metacognitive regulation (MR) is therefore of great importance in the clinical practice of clinicians,^11^^,^^14^ and its assessment is an important issue in both initial and continuing education.^11^

Although most of the literature focuses on learning, memory, and comprehension,^13^^,^^16^ there is a specific literature that concerns complex processes, such as clinical reasoning and problem-solving.^16^^,^^11^^,^^17^ Ackermann^16^ proposes a model with two main components of monitoring and control, which are detailed with several subcomponents. However, this model is generic to problem solving and, although applicable to clinical reasoning, it is not specific in the nature of tasks. Cloude^11^ proposes an approach centered on the clinical reasoning of physicians. Although he uses different terms (self-monitoring and self-regulation), these correspond to monitoring and control. However, his article does not provide any operational model more precise than the two major components. Johnson^17^ uses a taxonomy that employs yet different terms but proposes a highly operational model comprising several parameters to describe what they call self-assessment. The first parameter distinguishes between processes that are independent of a specific task and therefore relate more to general vigilance at a given moment or to the general representation that an individual may have of his or her abilities, and processes that are dependent on a task and therefore relate more to metacognitive regulation (MR).

When linked to a specific task, Johnson^17^ describes a second parameter, the timing, determined by two aspects: i) the moment at which the MR occurs in relation to the task, either just before (pre-task), during, or immediately after the task (post-task); and ii) the concurrent nature of MR when it occurs at the time the task is performed (lived), or retrospective when it occurs at a distance from the task performance, after a certain time, reliving the task performance by thinking back to it. These pre-during-post parameters apply to both concurrent and retrospective character of metacognitive regulation. The third parameter is the types of MR. On the one hand they defined self-observation, which is the recognition of one's thoughts, attitudes, or behaviors, and self-judgment, which refers to evaluating the accuracy or adequacy of one's thoughts, attitudes, or behaviors. These two parameters refer directly to monitoring.^16^ On the other hand, they defined self-reaction which concerns the planning, reinforcement, or implementation of a change in thoughts, attitudes, or behaviors. This corresponds to the definition of control.^16^ Thus, although Johnson uses the term self-monitoring, the three types of processes they define conceptually ultimately include monitoring and control, which constitute metacognitive regulation.

However, although this model is focused on healthcare professionals, and therefore not specific to PTs, it goes beyond clinical reasoning alone. The nature of the tasks is therefore not very structuring, especially so as some tasks could be grouped into identical gestural or mental activities.

To our knowledge, the literature on PTs' metacognitive regulation of clinical reasoning and its assessment has not been synthesized. Only the scoping review by Ziebart and MacDermid^18^ looks at PT reflexivity, which only covers the retrospective timing (according to Johnson^17^). It is also not specific to clinical reasoning and dates back six years. Moreover, an analysis of the search terms used in this review shows that only three terms related to reflexivity were employed, whereas the literature uses about twenty terms.^17^

The particular nature of PTs’ clinical reasoning suggests that the nature of regulation in the context of clinical reasoning probably has both shared and specific characteristics and purposes. Furthermore, the multiplicity of models and the variety of terms used suggest that there may be literature on the subject, but that it is likely scattered under several different terms. This observation also applies to the assessment of metacognitive regulation during CR. Given these elements and the importance of MR and its assessment in physical therapy, the aim of this study was to explore (1) the nature and purposes of metacognitive regulation in clinical reasoning activities in Physical Therapy, and (2) the breadth and diversity of methods and tools used to assess metacognitive regulation in clinical reasoning activities in Physical Therapy.

## Methods

To answer this exploratory question seeking to explore the nature of a concept within a population, a scoping review appears to be the appropriate methodology. A scoping review is a synthesis of the literature designed to explore the breadth or extent of literature on a specific topic, clarify a concept and its characteristics, map and summarize the evidence, and inform future research.^19^ We conducted this scoping review following the nine steps of the JBI guideline^19^ and the Prisma-Scr^20^ checklist (Appendix 1) and registered the protocol on Open Science Framework before the initiation of the search for evidence. The PPC Framework was used for this review: the population was PT and PT students; the concept was metacognitive regulation and the context was clinical reasoning which refers to a virtual or real care situation, experienced or not, in which clinical reasoning has been applied. No artificial intelligence was used in the completion of this work.

### Search Strategy

The literature search began on 04/01/2024, with the last update on 06/16/2024. The databases Pubmed, Google Scholar, PSYCinfo, EBSCO including CINAHL, and ERIC were queried. The search equations were intentionally broad and inclusive given the heterogeneity17 of terms used to describe metacognitive regulation (See more details and specific search strategies for each database in Appendix 2).

### Source of Evidence Selection

The selection focused on peer-reviewed articles and books in English and French, published after 2000. It was carried out by two researchers (YP/JN) in parallel and blinded, using the Covidence software, with predefined inclusion criteria (described in Appendix 3) and following three steps. All Discrepancies were resolved at each step by a discussion between the two reviewers based on the eligibility criteria. In case the conflict was not resolved, an independent arbitration plan with a third researcher was planned. Step 1: A pilot test on 25 randomly selected titles/abstracts, followed by comparison and discussion of selection discrepancies, and refinement of the criteria. Step 2: Selection based on title and abstract. Step 3: Selection based on the full text.

### Data Extraction

Data extraction was performed by two researchers (YP/JN) in parallel and blinded, using Dedoose software, following the same steps as for data selection. The extracted data focused on the year of publication, country, methodology, the nature and purpose of metacognitive regulation, and the tools and methods for assessing MR. To build the working conceptual framework, pre-established codes were used. In this scoping review, only task-dependent activities were considered. We used the framework developed by Johnson and colleagues,^17^ namely: the timing of MR (pre, during, post, concurrent, retrospective), the type of MR (self-monitoring, self-judgment, self-reaction). This represents what Jonhson calls self-monitoring and context-specific self-assessment. The type of task was also used, but Johnson's classification of tasks did not always seem appropriate, with some being very precise, such as a step in clinical reasoning, and others much broader, such as communication or management. This was a topic of discussion within the research team. In the end, according to Huhn's clinical reasoning definition, we chose a coding level based on the "skills" which constitute clinical reasoning, i.e. cognitive, psychomotor, and emotional (affective) activities. For the assessment tools and methods part, previous codes relating to the nature of metacognitive regulation have also been used to qualify these tools. Some specific codes were added to describe the nature of the assessment. For both nature and evaluation, a code named « other » was included to extract items that did not fit into one of the pre-established categories but were still relevant. An initial codebook was created based on the conceptual framework. Following step 3 of article selection by full text reading, the investigators updated the codebook. A selection of six articles that seemed to represent the diversity of the scope studied was made and the articles were coded. A dicussion between the investigators took place in order to adjust the codebook. The main addition was two tasks: i) the communication, which was found in certain articles and which seemed consistent in view of the constant interaction with the patient and ii) the « global management » as a task, which we considered to be the broadest task that could be described in detail and thus included in task-dependent metacognitive regulation. The six articles were then recoded with the adjusted codebook (Appendix 3). Then the extraction began. If a pre-established code was applicable to a given item, even just once, it was used.

### Analysis of the Evidence

The comparison and verification of the coding completed by the two coders was done. All discrepancies were resolved by a discussion between the two reviewers. In case of conflict, an independent arbitration was made by a third researcher. A synthesis based on a qualitative analysis for each code was conducted. This analysis aimed to structure the codes or data into different thematic categories. This was done for the nature of MR as well as for assessment tools. Data that did not fit into a predefined code but seemed relevant were the subject of an interpretative phase in order to consider the creation of a new theme.

An external PT, teacher, clinician and tutor for PT students in a hospital, with extensive knowledge of metacognition, was consulted at various stages of the research to ensure the consistency of the results with the literature and their relevance to Physical Therapy.

## Results

**Figure 1:**
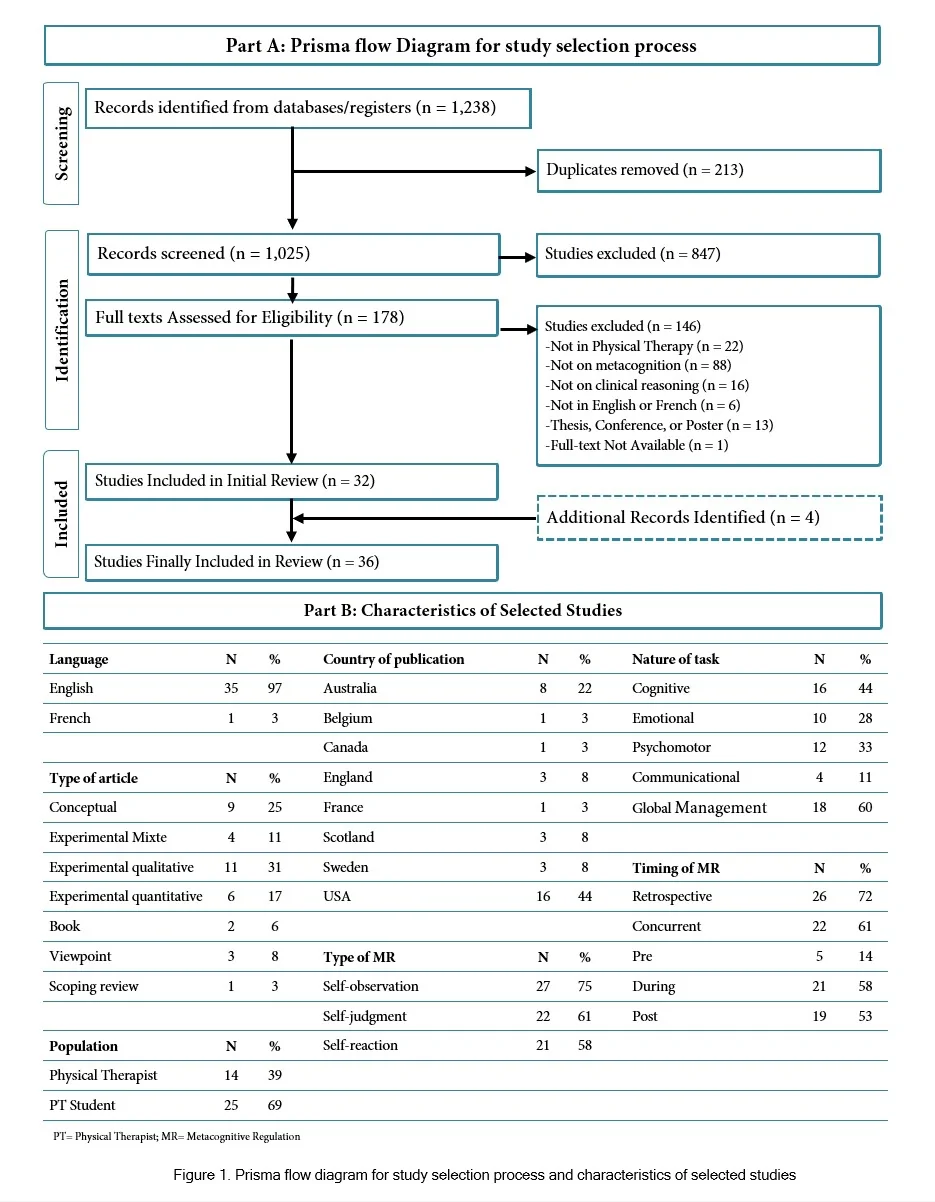
Prisma flow diagram for study selection process and characteristics of selected studies

After removing duplicates, the search reached 1,025 documents, of which 32 were included. Four articles were added following the latest search update, giving a total of 36 documents analyzed (Figure 1).

### Distribution of Publications

The publications analyzed (Figure 1) were published between 2000 and 2024, with a range of 1 to 5 per year. The majority of publications were American (n=16) and Australian (n=8), all in English (n=35) except one in French (n=1). Of the 36 publications, 20 were experimental, 12 were theoretical.  Regarding the population, the articles concerned students and professionals, but students were represented twice as often. The articles covered different levels ranging from novices to experts.

### Nature of Metacognitive Regulation

When analyzing the codes, we found that our operational framework was suitable for types and timing, task and purpose. However, some data did not fit and were classified as “other.” An inductive interpretive phase led to the creation of two themes: focal and interaction. We therefore structured the results of the nature of metacognitive regulation around five key characteristics: the types, the timing, the tasks, the focals and the interactions. Each concept is defined and operationalized below.

### Types of Metacognitive Regulation

The results fitted with Johnson’s^17^ three types of metacognitive activity: Self-observation ^18^^,^^21^^–^^26^(i.e. recognising one’s own thoughts, emotions, or actions), Self-judgment^9^^,^^18^^,^^21^^,^^22^^,^^24^^,^^27^^–^^39^(i.e. evaluating the adequacy or coherence of these elements) and Self-reaction ^9^^,^^18^^,^^22^^,^^25^^,^^28^^–^^33^^,^^37^^,^^39^^–^^42^(i.e. regulating or modifying reasoning or behaviour based on this evaluation).

### Timing of Metacognitive Regulation

The Johnson-based^17^ timing pre-codes were relevant and allowed us to classify the data, showing that MR is, on the one hand, concurrent ^9^^,^^18^^,^^21^^,^^23^^–^^26^^,^^28^^,^^31^^,^^37^^,^^41^^,^^43^ (i.e. around the time the task is performed) or retrospective ^9^^,^^18^^,^^21^^,^^26^^,^^29^^,^^30^^,^^37^^,^^39^ (i.e. at a distance from the task, which is then recalled); and on the other hand, pre-task ^9^^,^^33^^,^^34^^,^^41^i.e. before the task begins, during-task^18^^,^^22^^,^^23^^,^^25^^,^^28^^,^^30^^,^^37^^,^^39^^,^^41^^,^^43^^–^^45^i.e. while the task is running or post-task ^22^^,^^29^^,^^30^^,^^33^^,^^34^^,^^38^^,^^39^^,^^41^^,^^46^^,^^47^ i.e. just after the task is completed. Examples for type and timing: a PT preparing a treatment plan might anticipate possible patient responses (Self-judgment / Concurrent pre-task); during the session, they may adjust their manual technique in response to patient feedback (self-reaction / concurrent during task); and later, reflect on the preparation of their treatment plan (Self-judgment / retrospective pre-task) or reflect on their decision-making process during patient care (self-observation / retrospective during task).

### Task Characteristics

The classification of tasks presented in the methodology section was relevant.  In the cognitive task, MR targets clinical knowledge,^32^^,^^36^^,^^39^^,^^41^^,^^43^^,^^45^ clinical reasoning in general^18^^,^^22^^,^^23^^,^^25^^,^^29^^,^^32^^,^^36^^,^^37^^,^^39^^,^^40^^,^^45^^,^^48^ data interpretation,^32^^,^^48^ representation of situation,^18^^,^^39^^,^^45^^,^^48^ re-evaluation^18^^,^^23^^,^^37^^,^^39^ and cognitive bias awareness.^18^^,^^22^^,^^25^^,^^29^^,^^45^ The psychomotor task concerns reflection on movement and use of the body,^9^^,^^28^^,^^30^^,^^36^^,^^40^ gesture quality^28^^,^^31^^,^^32^^,^^33^^,^^39^^,^^45^ and tactile information processing.^28^^,^^36^^,^^40^^,^^45^ The emotional task involves identifying and regulating emotions that impact care decisions. ^18^^,^^22^^–^^24^^,^^25^^,^^39^^,^^40^^,^^48^ The communicational task relates to awareness and adjustment of verbal and non-verbal exchanges.^21^^,^^25^^,^^40^^,^^45^ The global management task is an appraisal of overall patient care effectiveness. For example: when a PT reflects on whether their mobilization technique was too forceful based on patient discomfort, this reflects a psychomotor dimension.

### Focals: Approach Used in Metacognitive Regulation

A first set of the data coded as “other” helped to bring out this first theme. Indeed, those data showed that MR was performed through a specific analysis grid, like a pair of glasses or a focal lens with a particular reading prism. We created a new key characteristic, named focal, that defined the analysis grid with which metacognitive regulation is achieved. Four focals were identified. The clinical focal concerns the relevance or effectiveness of reasoning for the specific clinical case. This raises the question: ‘’Are my processes effective and appropriate to the clinical situation and the patient?’’ This was the most prevalent focal. The ethical focal^22^^,^^36^ relates to the alignment with patient values, consent, and professional ethics. It raises the question: ‘’Are my processes adapted to professional ethics and the patient's values/beliefs/preferences?’’ The epistemologic focal questions the status and certainty of knowledge. It enables the PT to learn from and about his or her own practice, by questioning it.^18^^,^^40^^,^^45^ Uncertainty is clearly evident in the results,^22^^,^^23^^,^^25^^,^^29^^,^^34^^,^^38^^,^^39^ for which MR would be a compass^46^ for navigating through the complex and uncertain world.^18^^,^^25^^,^^27^^,^^37^^,^^46^^,^^49^  This comes down to asking the questions: ‘’What value can I attribute to the information/knowledge resulting from the processes I implement? What new knowledge have my processes enabled me to generate to enrich my scripts, and what is it worth?’’ Finally, the ontological focal^29^^,^^45^ raises the reflection on the practitioner’s identity and disciplinary congruence. This leads to the question: ‘’Are my processes based on foundations (models, knowledge, concepts...) which are in line with what Physical Therapy is? What does the analysis of my processes tell me about who I am as a practitioner?’’

### Intersections of Tasks and Focals

**Figure 2:**
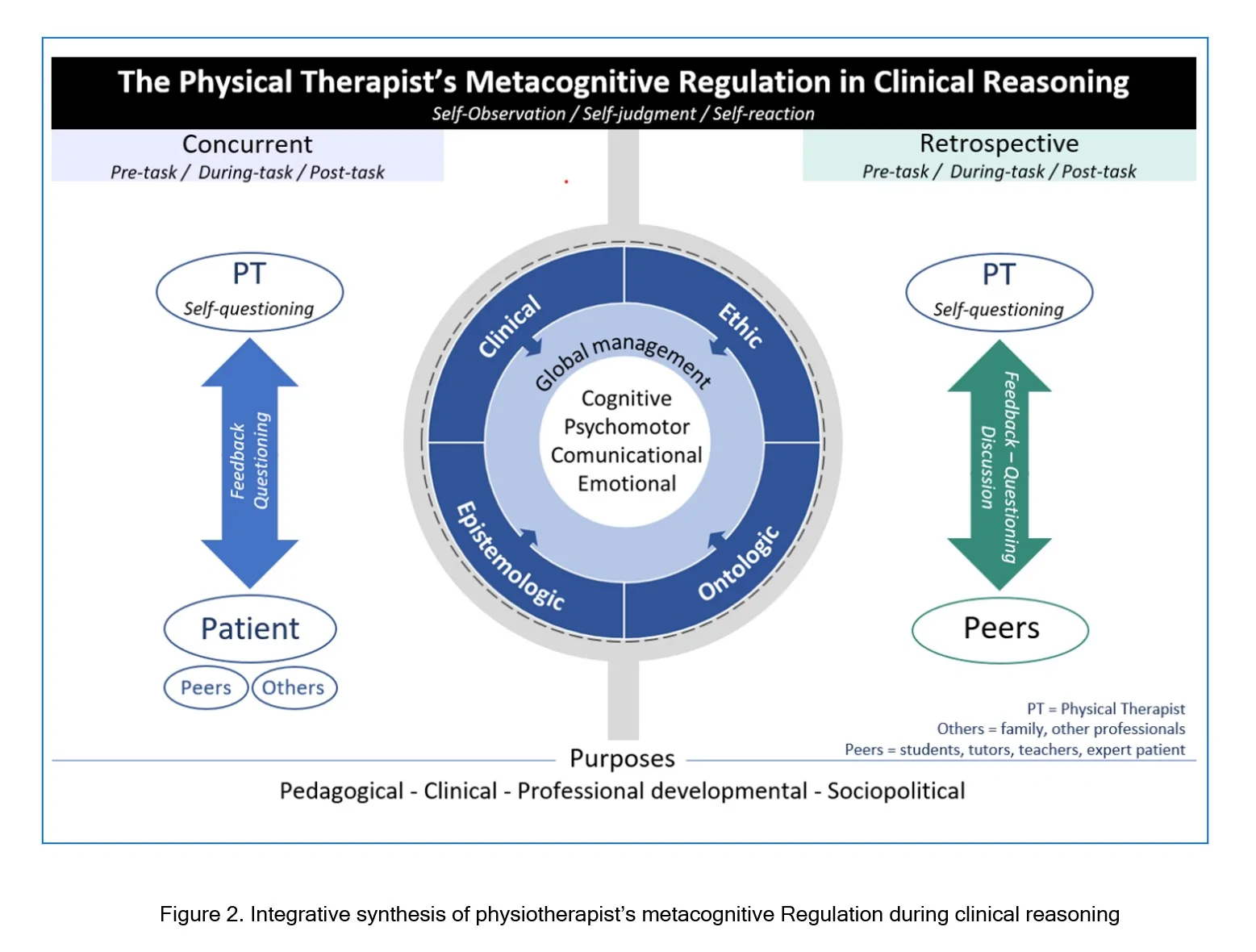
Integrative synthesis of physiotherapist’s metacognitive Regulation during clinical reasoning

We combined the five tasks and four focals into a matrix of intersections (see Table 1), allowing us to map how MR is engaged across domains and evaluative stances. Each entry in this table reflects a possible configuration of reflective inquiry. For example: a PT might assess whether their use of a technique aligns with the profession’s standards (psychomotor × ontological). A schematic diagram of the tasks and focals is proposed in Figure 2, located in the central part, surrounded by dotted lines.

#### Interactions: The Relational Nature of Metacognitive Regulation (Figure 2)

Another set of the data, coded as “other”, helped to bring out this second theme. Distinct from the conceptual intersections, interactions describe the dynamic, often dialogical nature of metacognitive regulation. They describe the persons in interaction during the PT's metacognitive regulation: themselves, the patient, the family, other professionals, and peers. Metacognitive regulation can occur in interaction with : (i) the PT themselves by self questioing^18^^,^^21^^,^^23^^,^^24^^,^^28^^,^^32^^,^^33^^,^^40^^,^^41^^,^^45^^,^^48^^,^^50^ ; (ii) the patient, for whom metacognitive regulation takes into account their responses and values ^21^^,^^25^^,^^29^^–^^31^^,^^40^but also the relationship with the patient^28^^,^^40^ or the patient's own regulation, for which the PT can become an educator of the patient's own metacognitive regulation abilities^28^^,^^31^^,^^49^ ; (iii) other external persons such as family, tutors, other professionals or peers.^33^^,^^40^^,^^44^^,^^45^^,^^52^

**Table 1:** Explanatory table of intersections between the dimensions and focal points of metacognitive regulation in clinical reasoning

**FOCALS**	**Clinical**	**Clinical**	**Ethical**	**Epistemological**	**Ontological**
TASKS	Is it appropriate and effective for this patient's situation?	Is it consistent with professional ethics and the patient's values?	-It allows me to consider that information/knowledge holds value.-It enriches my knowledge as new insights/scripts.	-It is aligned with the foundations of the Physical Therapy discipline.-It teaches me about my identity as a professional.
**Global management** Is my care approach	Is my overall care approach appropriate/effective for this patient'ssituation?	Is my overall care approach consistent with professional ethics and the patient's values?	Does reflecting on my overall care approach generate new knowledge?	Is my overall care approach aligned with the foundations of the Physical Therapy discipline and what does it teach me about my professional identity?
**Cognitive** Is my cognitive reasoning process	Is the cognitive process I engage in and the decision I make, appropriate and effective for the clinical situation? (Are the hypotheses I formulated relevant? Did I consider the variousavailable pieces ofinformation to make a decision?)	Are my reasoning and decision-making ethically acceptable (considering beliefs, ethics, values, etc.)? (Did I take the patient's values and preferences into account in my treatment proposal? Did I include the patient in the decision-making process?)	-What value can I assign to the information or conclusions produced by the reasoning I have just undertaken?-What does reflecting on my process and decisions teach me as new knowledge? (Did I conduct unreliable tests, and did I place too much weight on their results? What have I learned regarding the association of different clinical signs in this situation? To what extent has my epistemic stance influenced my decisions?)	-Does my cognitive reasoning adhere to professional standards?-What does analyzing my reasoning teach me about who I am and my reasoning style? (Do I utilize standards such as Evidence Based Practice, Bio-psycho-Social, and International Classification of Functioning? Do I tend to close my reasoning too quickly when faced with a patient having a pathology I am familiar with?)
**Psychomotor** Are my actions	Is the action I perform appropriate and effective for the clinical situation and patient? (Does my action produce the desired effect? Is it effective? Is it adapted to the patient's pain?)	Is the action I perform ethically acceptable in light of the patient's values? (Has my action been subject to inform consent, does it respect the patient's beliefs, and is this action indeed accepted by the patient?)	-What value can I assign to the information collected through the action I have just performed?-What new knowledge does the action provide me? (Does my action, as performed, lead me to believe that the tactile information derived from it holds value? What has the execution of this action taught me regarding the refinement of my movement scripts?)	-How does the action I perform fit within the discipline? Is it Physical Therapy?-What does analyzing my reasoning teach me about who I am and my practice style? (Is my action consistent with the Physical Therapy's recommendations? Is performing this action considered within the scopen of Physical Therapy? I realize that I only engage in hands-off techniques without ever employing passive techniques.)
**Emotional **Are my emotions and reactions	What are my emotions, and are my reactions appropriate for the clinical situation? (I perceive that I am stressed due to my inability to find a solution,leading me to retestcontinuously withoutmaking a decision.)	In response to these emotions, are myreactions ethicallyappropriate in light of my professional status and the patient's values? (I perceive that I am stressed, which results in my frustration at the patient's refusal of a technique, although it is recommended by the literature.)	-How will the emotion I experience impact the value of the information I collect or the conclusions I draw?-What does analyzing my emotions teach me as new knowledge? (I perceive that I am stressed due to the patient's pain, which alters the value I assign to the information,potentially affecting my prioritization. Out of fear, I exhibit a selection bias that lead me to emphasize information justifying the use of passive techniques.)	How does the emotion I experience and the management of it inform my emotional regulation as a professional? (When I am stressed, I tend to narrow my reasoning, propose multiple decision pathways to the patient, and observe which one they seem to favor. I then guide them toward the option they prefer, ultimately leaving the decision to them without positioning myself.)
**Communicative** Is my communication	Is my communication appropriate for the clinical situation/patient? (Considering language level, message compliance,reception of the message.)	Is the communication I engage in ethically acceptable? (Considering therapeutic distance, professional language, informed consent, etc.)	What value can be assigned to the information produced by the communication I have just conducted? (Are the questions overly directed or closed? What is important to communicate to the patient?)	How does my communication and its management inform my understanding of the professional I am? (Do I easily use informal language? Do I maintain minimal professional distance with peers, and do I allow the patient enough opportunity to speak.)

### Operational Summary

The five key characteristics type, timing, task, focal and (when applicable) interaction, provide a comprehensive and situated understanding of MR in PT reasoning. Definitions and examples are synthesized in Table 2.

### Purposes of Metacognitive Regulation

In this section, we synthesize the purposes of MR as they are reported in the literature. The results show that the two expected codes (learning and quality of care) were found, but several data points did not fit these codes. After an inductive phase of data analysis, we organized codes into four overarching categories: pedagogical, clinical, professional developmental, and sociopolitical purposes (Fig.2): Each category reflects a different scope of impact—from the immediate patient encounter to broader issues of identity and transformation within the profession.

Pedagogical Purpose. Metacognitive regulation is described as a lever for learning^9^^,^^18^^,^^22^^,^^34^^,^^41^^,^^45^^,^^50^ both for students and practitioners. It facilitates the development of clinical reasoning skills^9^^,^^22^^,^^28^^,^^29^^,^^31^^,^^32^^,^^34^^,^^36^^,^^41^^,^^52^^,^^53^ supports the transition from declarative to procedural knowledge, and enhances the ability to self-assess and regulate learning strategies.

Clinical Purpose. Authors emphasize that at the point of care, MR allows practitioners to adjust their reasoning in real time, improving the safety, quality and equity of care,^18^^,^^23^^,^^29^^,^^36^^,^^41^^,^^47^^,^^50^ and to navigate uncertainty.^22^^,^^25^^,^^28^^,^^30^^,^^40^^,^^41^^,^^46^ It supports safer, more responsive, and individualized care.

Professional Development and Identity Purpose. Based on the data provided, MR contributes to the formation and evolution of professional identity. It fosters epistemic vigilance, ethical sensitivity, and ontological alignment with the values and foundations of physiotherapy.^51^ Through ongoing self-questioning, practitioners consolidate their identity as reflective and autonomous professionals.^44^

Sociopolitical and Emancipatory Purpose. Authors highlight the transformative potential of MR beyond individual or clinical improvement. MR can be a tool of emancipation^29^^,^^44^helping practitioners and students recognize implicit norms,^40^^,^^50^ challenge established power dynamics, and advocate for equity in care. To make this purpose more tangible, we refer to it as the critical emancipatory function of MR. It enables professionals to distance themselves from taken-for-granted assumptions and to act as agents of change within institutions and educational systems, enabling the standard of the profession to be raised.^27^

The aggregation of all identified results provides an integrative synthesis of the PTs metacognitive regulation during clinical reasoning (Figure 2).

### Evaluation of Metacognitive Regulation

Only four studies were selected.^29^^,^^33^^,^^34^^,^^50^ Five other articles dealing with evaluation by assessment methods based on self-questionnaires were found but not included as they were not task-dependent. The assessment of metacognitive skills is mentioned by the authors as having a formative or summative purpose. There appears to be no specific framework for developing grading criteria.^52^ The four studies were summative (quantitative) in their assessment methods but none reported a qualitative or psychometric evaluation of their method. Two main categories were found.

### Assessment Based on Qualitative Data

These assessments are based on qualitative data collected by students about situations they have experienced in placement situations. Evaluators assign scores based on this data. Such data may come from i) blogging activities between peers in which the quality and content of feedback or discussions are assessed33, ii) the use of a guide such as the critical reflection assessment task^50^ based on placement situations and marked out of 100 with subjective markers, iii) a guided personal reflective report,^29^ on a situation experienced during the placement, which accounts for 20% of the placement mark. The tasks assessed were cognitive, emotional, psychomotor and global management. Communication was not specifically addressed.

### Assessment Based on Quantitative Data

Only one study^34^ concerns this category. It quantifies MR by measuring calibration and level of certainty. Calibration is the correlation between performance judgment and actual performance. According to Prokop,^34^ calibration measurements should be carried out regularly throughout the Physical Therapy curriculum, in particular by adding prediction (before the task) and postdiction (after the task) sections to each written or practical assignment or examination. Prokop^34^ also highlights the importance of measuring certainty, which she defines as "a subjective estimate of expected performance". Students are asked to rate the confidence they have in their answers. The correlation between the level of confidence and the actual outcome of the question can then be quantified. These assessments were concurrent, pre or post-task, focused on self-judgment, cognitive or psychomotor tasks and based on lived or fictitious situations (written cases).

## Discussion

This scoping review aimed to explore (1) the nature and purposes of metacognitive regulation in clinical reasoning activities in Physical Therapy, and (2) the breadth and diversity of methods and tools used to assess metacognitive regulation in clinical reasoning activities in Physical Therapy. This work identified 36 studies. The population covered in the articles consisted mainly of students and professionals, ranging from novices to experts. Transferability therefore seems possible for these two populations. The diversity of the countries of origin, mainly from the United States and Australia and then from, Sweden, Scotland, England, France, Canada and Belgium, suggests that transferability is likely to be significant in Anglo-Saxon and some European countries.

**Table 2:** Operational summary- key characteristics of PT metacognitive regulation in clinical reasoning

Operational Summary	
Definition of key characteristics	
Type	Defines the processes of metacognitive regulation as self-observation, self-judgment, andself-reaction
Timing	Defines when metacognitive regulation takes place in relation to the task, i.e. concurrent orretrospective, and specifies whether it is pre-task, during-task, or post-task.
Task	Defines the particular nature of clinical reasoning task on which metacognitive regulation isperformed: cognitive, psychomotor, emotional, communicational, or global management
Focal	Defines the analysis grid with which metacognitive regulation is achieved: clinical, ethical,epistemological or ontological
Interaction	Defines the persons in interaction during the PT's metacognitive regulation: themselves, the patient, the family, other professionals and peers.
Application of key characteristics	
Example 1	Just after the end of a session with a patient (timing), the PT wonders whether their diagnosticcategorization (interaction / task) has not been biased (type) by a premature closure of reasoning, and whether this has globally altered the session (focal).
Example 2	One week after the end of their placement (timing), during an analysis of professional practices workshop with two other students (interaction), a PT student remembers the gestures (task) he performed (type) during his treatment of the patient (timing), asking himself if he had paid attention to the patient's reactions (interaction) in order to adjust the intensity of his gestures (focal).

### Nature of Metacognitive Regulation

The concept of "nature" as initially described in our framework now unfolds through the articulation of five distinct key characteristics: type, timing, task, focal and interaction.

The characteritics of type and timing fitted well to those described by Johnson^17^ which could reinforce the relevance of this taxonomy. The different types of MR were well represented (from 58 to 75%). All timings were also fairly well represented (between 53 and 72%) in the literature except for the pre-timing, which was underrepresented (14%). The tasks were mainly on global management (60%) and cognitive (44%), then psychomotor (33%) and emotional (28%) and least communicational (11%). The high density of the psychomotor dimension is surprising in comparison with the literature, which is much more focused on the cognitive, but it is not surprising in view of the clinical reasoning and the singularity of the movement scripts of PTs. Furthermore, the PT seen as an educator of the patient's psychomotor MR is an element which has not been found in the preliminary research on metacognitive regulation, including for other professions. The tasks created, which reflect the various domains within which metacognitive regulation operates, turn out to be similar to those in Guyet's^40^ model and have been independently identified by other authors. At this point, MR is well represented in the article included for the type, timing and task, except for pre-task timing and communicational task.

The data enabled the creation of clinical, ethical, epistemological, and ontological focals, which are all consistent with the literature on clinical reasoning.^7^^,^^9^^,^^54^^,^^55^ The clinical focal was mainly represented. Also not expected a priori, epistemologic focal that emerges from the results is not surprising as uncertainty is an element intrinsically present in physical therapy^56^ and linked to clinical reasoning.^55^ Furthermore, this ties in with the concept of personal epistemology.^55^^,^^57^

Concerning the Interaction characteristic, results underline that MR is not only an internal process focused on the self. Our analysis shows that it is deeply relational and interactive. Whether through real-time feedback, PT-patient exchanges, or retrospective peer discussions, MR is often co-constructed. The identification of interactions complements the intersections between tasks and focals, revealing a dynamic space where clinical reasoning seems to be continuously negotiated and refined.

### Purposes of Metacognitive Regulation

This review identifies four primary purposes of MR engagement: pedagogical (learning and consolidation), clinical (adaptation and safety), professional developmental (identity and responsibility), and sociopolitical (emancipation and transformation). The first two were expected and underline the role of MR in learning and clinical reasoning developement. This could be explained by the cognitive dimension, which is fundamental to the organization of knowledge and reasoning in physical therapy, as well as the other complementary tasks.

The sociopolitical purpose seems to be congruent with clinical reasoning development orientations based on foresight for the future of healthcare, as it can be described in Australia or the United States.^55^ This suggests that metacognitive regulation functions not only as a cognitive-regulatory mechanism, but also as a transformative force within education, care, and the profession itself. The data suggest that the four purposes are not mutually exclusive; rather, they are often interwoven. For instance, the development of clinical reasoning through metacognitive training may simultaneously reinforce professional identity and ethical sensitivity. Similarly, reflective interactions with peers could contribute to all four purposes depending on context.

Finally, several articles ^9^^,^^24^^,^^25^^,^^29^^–^^32^^,^^35^^,^^41^^–^^46^^,^^50^ establish links between these purposes, the tasks and focals, reinforcing the relevance of linking them together in the integrative model.

The integration of all these key characteristics and purposes in a comprehensive model, associated with an operational table of intersections between task and focal points is the main contribution of this study concerning the nature of MR. Yet, it remains consistent with the various conceptual models used in the literature, such as Schön's^9^^,^^22^^,^^26^^,^^28^^,^^30^^,^^33^^,^^37^^,^^39^^,^^42^^,^^44^^,^^46^^,^^47^ and the models combining Schön's with cognitivist,^23^^,^^45^ motivational^41^ or vocational didactic theories.40 This layered understanding of MR could be an avenue for future reflection and research for the development of PT education and practice.

### Evaluation of Metacognitive Regulation

The results of the evaluation, although very limited, showed diversity. Most types, timing, and tasks were covered but mostly once. Only communication was not specifically included which is nevertheless essential in PT. Despite this diversity, it appears that the proposals remain much more limited than for other professions and especially with regard to measurement. Physical Therapy seems to fall short of the possibilities found in the literature.^11^^,^^58^^,^^59^^,^^60^ This shows that evaluation is probably underexploited in PT, especially given the small number of articles and the lack of qualitative or psychometric evaluation of the methods used. This last aspect limits the interpretation and utilization for certification purposes. Evaluating metacognitive regulation remains a challenge, especially when attempting to capture its multidimensional and situated nature. According to the five key characteristics presented above, existing methods do not seem to adequately account for the subtle interplay between tasks and focals or the dynamic nature of interaction.

### Limitations and Future Directions

This scoping review should be considered as having a descriptive purpose, associated with ideas for reflection on its application in the field. It is not intended to be evaluative or prescriptive in terms of the attitude to be adopted. This review is limited by the heterogeneity of definitions and conceptualizations of MR across studies, by the use of Google Scholar which has certain reproducibility flaws, by the selection of the literature published after 2000, by the exclusion of grey literature and, by the possible over-representation of certain codes due to their inclusion if present only once. Despite the particular attention paid by the research team, potential biases related to selection and data interpretation cannot be ruled out, and likewise, the dual screening inter-rater agreement statistics has not been assessed. Additionally, although our framework was developed on the basis of a robust qualitative synthesis, further empirical research is needed to test its applicability in educational and clinical settings.

The integrative model created in this study could offer an opportunity to design future research on more targeted pedagogical interventions, such as integrating case-based discussions that explicitly require epistemological or ontological reflection, simulation debriefings that stimulate emotional and psychomotor awareness; or multi-dimensional assessment with triangulation of data sources (e.g., written reflections, clinical observations, verbal reasoning during simulations) aligned with the intersections and interactions describes in the model. Future studies should examine how training programs can be aligned with this integrative model, and how evaluation tools might capture not only the presence of metacognitive regulation, but its quality, depth, and contextual sensitivity. Its transferability to other health professions could also be the subject of future research.

## Conclusions

Through this scoping review, we found that metacognitive regulation in physical therapists’ clinical reasoning is a complex, multidimensional, and interactional process. It engages various aspects of professional practice, from technical precision to ethical reflection and professional identity formation. The integrative model, created from the data, provides a structured and comprehensive lens through which to understand metacognitive regulation in educational and clinical settings. This work helps set the stage for future project srelating to education or evaluation, grounded in physical therapy clinical reasoning, and ultimately, foster a rich and meaningful metacognitive engagement for developing reflective, responsive, and socially conscious practitioners.

### Acknowledgments

The authors wish to thank Florie Goulard for her advice and comments at various stages of the process. This work was partly supported by the Association Nationale Formation des Hospitaliers Océan Indien.

### Conflict of Interest

The authors declare that there is no conflict of interest.

## Supplementary material

Supplementary file 1 Appendix 1. Preferred reporting items for systematic reviews and meta-analyzes extension for scoping reviews (PRISMA-ScR) Checklist

Supplementary file 2 Appendix 2. Search strategy

Supplementary file 3 Appendix 3. Source findings and data extraction instrument
