# Supplementary material for: Metacognitive regulation in physical therapists’ clinical reasoning: a scoping review
Source: Int J Med Educ. 2026 Jun 3;17:61–76. doi: 10.5116/ijme.6a0c.4c0b (PMC13563282; doi:10.5116/ijme.6a0c.4c0b)
Supplement: Supplementary file 2 [file ijme-17-61-S2.pdf]

Appendix 2

Search strategy

Databases

PubMed - Google Scholar - PsychINFO - EBSCO (including CINAHL, Academic Search Complete, eBook Collection, SportDiscus, Ergonomics Abstract) - ERIC.
Due to the exploratory nature of a scoping review and given that the topic can encompass clinical approaches as well as cognitive, behavioral, psychological, and social approaches, which are more relevant to the humanities, highly specialized clinical websites such as PubMed did not seem to cover the field adequately, unlike Google Scholar, which provided broader coverage.

Search queries

For PubMed
(("clinical reasoning") AND ((physiotherapy) OR ("physical therapy"))) AND ((((((((((((metacognition) OR (metacognitive)) OR ("self-monitoring")) OR ("self-assessment")) OR ("self-awareness")) OR ("self-regulation")) OR (reflexive)) OR (reflexivity)) OR ("self-reflection")) OR (reflection)) OR ("self-reaction")) OR ("self-judgment")) OR ("self-observation"))

For Others
(physiotherapy OR "physical therapy") AND (metacognition OR metacognitive OR "Self-monitoring" OR "self-assessment" OR "self-awareness" OR "self-regulation" OR reflexive OR reflexivity OR "self-reflection" OR reflection OR "self-reaction" OR "self-judgment" OR "self-observation") AND "clinical reasoning")

Strategy

We processed as a first step an initial limited search using PubMed and Google Scholar. We selected articles by title and abstract that correctly corresponded to the scope. We analyzed the key words used. A second search using all identified keywords and index terms was proceeded across all included databases.

The search validation procedure was made by testing queries that

1) include major articles identified at the first search stage
2) include articles whose titles seem to match our criteria

- For Google Scholar, the "journal article" option was ticked. That reduced the results from nearly 8,000 to 1,731. Only the first 1,000 articles were downloaded, in accordance with the technical constraints of the search engine.
- For EBSCO and ERIC, the "peer-reviewed" and French-English language options have been ticked. Grey literature (dissertations, theses, conferences) was excluded. Indeed, due to the large number of terms used in the literature for the concept, it seemed more appropriate to retain the variety of terms but to limit the literature to published books and articles rather than limit the variety of terms and include grey literature.

A complementary search, as an update was performed just before data charting.
Deduplication has been done in Covidence software, including an analytical check by the researchers before excluding the document.
The limitation to French and English was due to the languages mastered by the researchers, without the need for translators.
