# Supplementary material for: Metacognitive regulation in physical therapists’ clinical reasoning: a scoping review
Source: Int J Med Educ. 2026 Jun 3;17:61–76. doi: 10.5116/ijme.6a0c.4c0b (PMC13563282; doi:10.5116/ijme.6a0c.4c0b)
Supplement: Supplementary file 3 [file ijme-17-61-S3.pdf]

Appendix 3

Source findings and data extraction instrument

**Scoping Review detail**	
Title	Metacognitive Regulation in Physical Therapists' Clinical Reasoning: a Scoping Review
Objectives	1) Explore the nature and purposes of metacognitive regulation in clinical reasoning activities in Physical Therapy<br>2) Explore the breadth and diversity of methods and tools used to assess metacognitive regulation in clinical reasoning activities in Physical Therapy
**Inclusion/Exclusion Criteria**	
Population	Physical Therapists (PTs) or PTs students
Concept	Focused on metacognitive regulation
Context	- Concerns clinical reasoning<br>- Describes a model or characteristics of metacognitive regulation (MR) OR/AND describes a method/tools for assessing MR
**Exclusion Criteria**	- In a language other than English or French<br>- Focused on clinical reasoning rather than metacognitive regulation<br>- Focused on meta-memory / metacognitive regulation on learning without clinical reasoning<br>- Not focused on Physical Therapist or PT student
**Evidence Sources Characteristics**	- Year<br>- Country<br>- Study population<br>- Type of article : (Conceptual article, Qualitative experimental article, Quantitative experimental article, Mixed experimental article, Viewpoint article, Book, Other)
**Part 1 - Nature of Metacognitive Regulation**	
**Nature of Task**	- Cognitive<br>- Communicative<br>- Psychomotor<br>- Emotional<br>- General PEC<br>- Task-dependent or task-independent (if independent exclusion)<br>- Other
**Task Timing**	- Concurrent<br>- Retrospective<br>- Pre<br>- During<br>- Post
**Type of Metacognitive Regulation**	- Self-observation<br>- Self-judgment<br>- Self-reaction
**Other**	Other items that do not fit into one of the pre-established categories

**Purpose of Metacognitive Regulation**	- Learning<br>- Quality of care<br>- Other
**Part 2 - Assessment of Metacognitive Regulation**	
**Nature of task assessed**	- Cognitive<br>- Communicative<br>- Psychomotor<br>- Emotional<br>- General PEC<br>- Task-dependent or task-independent (if independent exclusion)<br>- Other
**Timing of task assessed**	- Concurrent<br>- Retrospective<br>- Pre<br>- During<br>- Post<br>See note* for precision
**Type of metacognitive regulation**	- Self-observation<br>- Self-judgment<br>- Self-reaction
**Nature of assessment**	- Quantitative or Qualitative data<br>- Quantitative or qualitative Assessment<br>- Summative or Formative purpose<br>- Assessment setting/Place of performance<br>- Nature of the Situation (used for assessment)
**Other**	Other items that do not fit into one of the pre-established categories

**Note: Johnson's study highlights the difficulty of differentiating these two levels of timing. The principle was to: 1) Define whether regulation is performed by the person more than 4 hours after the task (the duration proposed by Johnson) to determine if it is concurrent or retrospective. 2) Analyze whether regulation occurs Pre, During, or Post the task.*
